# Evaluation of GFAP and phosphorylated tau correlation in human blood using magnetic bead‐based electrochemical sensor

**DOI:** 10.1002/alz70856_102564

**Published:** 2025-12-25

**Authors:** Hana Cho, Kyoung‐Ryul Lee, Seongeun Jeong, Sang‐Heon Park, Soo Hyun Lee

**Affiliations:** ^1^ Korea Institute of Science and Technology, Seoul, Korea, Republic of (South); ^2^ KIST school, University of Science and Technology, Seoul, Korea, Republic of (South)

## Abstract

**Background:**

Blood biomarkers are regarded as a method for the diagnosis, prognosis, and monitoring of Alzheimer's disease (AD). Amyloid‐beta (Aβ) and phosphorylated tau (*p*‐tau) are widely used blood biomarkers; nonetheless, it is essential to explore additional promising candidates for the validation of pathological mechanisms and the reliability of clinical data. Glial fibrillary acidic protein (GFAP) has the potential to reflect and predict worsening disability in individuals with degenerative diseases. We aimed to measure GFAP and *p*‐tau levels in human blood and evaluate the correlation.

**Method:**

Magnetic beads were coated with a primary antibody that was specifically bound to the antigen. For the GFAP tests, beads were incubated with 3 groups from: 1) normal control (NC, *n* = 5), 2) mild cognitive impairment (MCI, *n* = 5), and 3) Alzheimer's disease (AD, *n* = 5). For *p*‐tau levels measurement, all samples were bound to secondary antibodies such as O‐glycosylated tau (O‐g tau) and *p*‐tau. We compare GFAP and *p*‐tau impedance change rates to diagnose NC/MCI/AD and discuss the correlation between the two AD biomarkers.

**Result:**

In the NC group, both GFAP and *p*‐tau exhibited low rates of impedance change. Patients with MCI showed a slightly high rate of impedance change compared to the NC. We established a cut‐off value to enhance the diagnostic reliability of NC and MCI. The AD patient exhibited the highest rate of impedance change, about double that of NC. The enhanced reliability of multi‐biomarkers was substantiated through a comparative analysis of the area under curve (AUC) value of a single biomarker against the AUC value derived from multi‐biomarkers.

**Conclusion:**

We evaluated the correlation between GFAP and *p*‐tau levels in human blood using a magnetic bead‐based electrochemical sensor for AD diagnosis. We could determine the extent of astrocyte activation in individuals with NC, MCI, and AD using a GFAP protein. Moreover, the cross‐validation of the impedance change rates indicates that the AUC value for the multi‐biomarkers is superior to that of a single biomarker. Successful validation of a point‐of‐care platform for blood biomarkers such as GFAP and *p*‐tau could increase the diagnostic reliability of AD, which could forecast the prognosis of AD.

